# Combination of metronomic cyclophosphamide and dietary intervention inhibits neuroblastoma growth in a CD1-nu mouse model

**DOI:** 10.18632/oncotarget.7929

**Published:** 2016-03-05

**Authors:** Raphael Johannes Morscher, Sepideh Aminzadeh-Gohari, Cornelia Hauser-Kronberger, René Günther Feichtinger, Wolfgang Sperl, Barbara Kofler

**Affiliations:** ^1^ Laura Bassi Centre of Expertise-THERAPEP, Department of Pediatrics, Paracelsus Medical University, 5020 Salzburg, Austria; ^2^ Department of Pathology, Paracelsus Medical University, 5020 Salzburg, Austria; ^3^ Department of Pediatrics, Paracelsus Medical University, 5020 Salzburg, Austria; ^4^ Division of Medical Genetics, Medical University Innsbruck, 6020 Innsbruck, Austria

**Keywords:** neuroblastoma, ketogenic diet, glucose, metronomic cyclophosphamide, anti-angiogenic

## Abstract

**Background:**

*MYCN*-amplification in high-grade Neuroblastoma (NB) tumors correlates with increased vascularization and therapy resistance. This study combines an anti-angiogenic approach with targeting NB metabolism for treatment.

**Methods and Results:**

Metronomic cyclophosphamide (MCP) monotherapy significantly inhibited NB growth and prolonged host survival. Growth inhibition was more pronounced in *MYCN*-amplified xenografts. Immunohistochemical evaluation of this subtype showed significant decrease in blood vessel density and intratumoral hemorrhage accompanied by blood vessel maturation and perivascular fibrosis. Up-regulation of VEGFA was not sufficient to compensate for the effects of the MCP regimen. Reduced Bcl-2 expression and increased caspase-3 cleavage were evident. In contrast non *MYCN*-amplified tumors developed resistance, which was accompanied by Bcl-2-up-regulation. Combining MCP with a ketogenic diet and/or calorie-restriction significantly enhanced the anti-tumor effect. Calorie-restricted ketogenic diet in combination with MCP resulted in tumor regression in all cases.

**Conclusions:**

Our data show efficacy of combining an anti-angiogenic cyclophosphamide dosing regimen with dietary intervention in a preclinical NB model. These findings might open a new front in NB treatment.

## INTRODUCTION

Neuroblastoma (NB) represents the most common extracranial solid childhood cancer. Marked biological and clinical heterogeneity, pose strong challenges to optimizing therapeutic interventions for individual cases. Among others biological risk factors include *MYCN* status, tumor histology, cancer cell DNA content and defined segmental chromosomal aberrations [1–5]. In combination with historical clinical data, tumor biology allows clinicians to guide therapy by stratifying patients into internationally accepted risk groups [6, 7]. In low- and intermediate-risk groups, with overall survival rates above 90%, recent studies have been focusing on reducing therapeutic toxicity. For patients in the high-risk group, however, improving treatment efficacy is still central with overall survival rates close to 50% despite multimodal therapy protocols [1].

With regard to cellular metabolism NBs share the characteristic reprogramming to high glucose uptake recently added to the hallmarks in cancer [8–10]. This preferential utilization of glucose via aerobic glycolysis even under sufficient oxygen to shunt pyruvate into oxidative phosphorylation (OXPHOS) pathway, is commonly described as Warburg effect [11, 12]. Whereas defects in single OXPHOS subunits can be a direct cause of the Warburg effect [13–15], other cancers show a general pattern of low OXPHOS activity [16–19]. In NB, both primary tumor tissue and xenografts display generalized low mitochondrial respiratory chain activity [20, 21]. This metabolic feature might indicate a dependency on glucose not only for anabolic processes, but also for energy production. Based on this theory, we recently reported a preclinical study showing significant tumor growth inhibitory effects of dietary intervention in a NB xenograft model [21]. In that study we observed that a mild ketogenic diet (KD) and/or calorie restricted (CR) diet altered blood metabolic parameters (reduced glucose and increased beta-hydroxybutyrate levels) and induced significant growth inhibition of NB xenografts. Detailed evaluation of mitochondrial OXPHOS parameters showed no adaptive response in the NB tumors [21].

These growth inhibitory effects are in line with preclinical data on cancers of the central nervous system suggesting that CR-KD might constitute an adjuvant approach in cancer therapy [22–25]. Effects of CR and/or KD in diverse preclinical cancer models have recently been reviewed [26, 27]. Anecdotal case reports of cancer patients treated with CR-KD support a possible inhibitory effect on tumor growth, but comprehensive preclinical and clinical evaluation is lacking [28–30]. Pilot studies of KD in adult patients showed that it might be tolerated in patients with advanced cancers [31–33].

Because targeting cancer metabolism is complementary to current treatment strategies for NB, it could potentially open a new front against this tumor entity. The current study therefore aims to investigate the combination of KD and/or CR diet with a low-dose metronomic cyclophosphamide (MCP) chemotherapy regimen. MCP has been shown to effectively inhibit neoangiogenesis in xenograft models of different tumor entities [34–36] and is under clinical evaluation [37–40]. We hypothesize that concurrent targeting of tumor vascularization and cancer cell metabolism might constitute a cooperative therapeutic approach.

## RESULTS

### Inhibition of NB tumor growth by MCP in combination with dietary intervention

**Figure 1:**
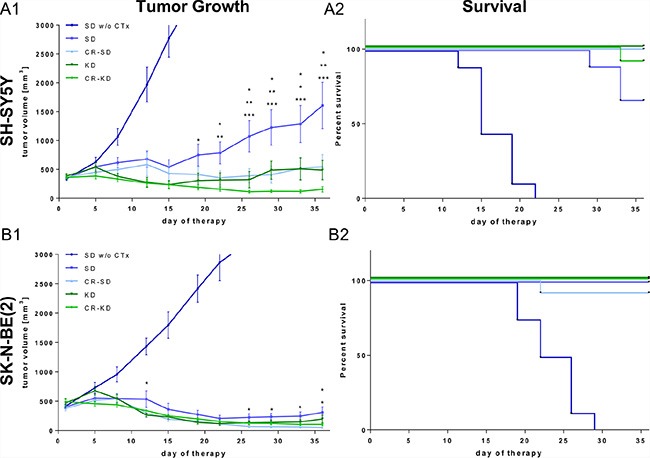
Dietary intervention enhances the growth inhibitory effect of MCP on NB xenografts After establishing tumors, mice were randomized to therapy and control groups as indicated. For xenografts of both cell lines the MCP regimen significantly inhibited tumor growth compared to the SD group w/o CTx (*p* < 0.001). (**A1**) SH-SY5Y and (**B1**) SK-N-BE(2) tumor growth curves. Data points represent mean values ± SEM of the corresponding therapy group (*n* = 8–12). (**A2**) and (**B2**) show Kaplan–Meier survival curves for mice with SH-SY5Y and SK-N-BE(2) xenografts respectively. Survival was significantly prolonged in all therapy groups when compared to the SD group w/o CTx (*p* < 0.001). The effect of dietary intervention on tumor growth was evaluated by comparing diet groups to the corresponding SD on MCP. Significance levels are given for each dietary intervention group compared to SD on MCP and are stacked from the group with lowest to highest tumor volume. Statistics: ANOVA (*p* < 0.05) followed by two-tailed Dunnett's test correcting for multiple comparisons; *p ≤ 0.05; **p ≤ 0.01; ***p ≤ 0.001. Differences in survival were determined in a univariate analysis with the log-rank test. Death is coded: tumor volume above 3000 mm^3^, tumor ulceration or impaired health condition. Abbrev.: SD, standard diet; CR, calorie restriction; KD, ketogenic diet; w/o CTx, without chemotherapy; MCP, metronomic cyclophosphamide.

In an effort to exploit a potential synergism of targeting vascular supply and cancer cell metabolism, we chose to combine dietary intervention with MCP. In the presented model, MCP induced significant growth inhibition (*p* < 0.001) of NB xenografts of both cell lines tested from day 9 onward and extended survival (*p* < 0.001) compared to the corresponding standard diet group without MCP (SD group w/o CTx; Figure 1). Intriguingly on the metronomic dosing protocol, growth inhibition of SK-N-BE(2) tumors was more pronounced versus that of tumors of the reportedly more chemotherapy sensitive SH-SY5Y cell line [41–43]. In addition to the chemotherapy-induced growth reduction, SH-SY5Y tumors showed a significant additional growth inhibition in all three dietary intervention groups (Figure 1A1). On day 36, tumor volume in the SD group was significantly greater compared to that of the calorie restricted-standard diet (CR-SD) group (*p* < 0.05), the KD group (*p* < 0.01) and the CR-KD group (*p* < 0.001). In the SK-N-BE(2) xenografts tumor growth was significantly inhibited in the restricted diet groups (Figure 1B1) CR-SD (*p* < 0.05) and CR-KD (*p* < 0.05). Based on the strong effect of MCP on SK-N-BE(2) tumor growth, observed absolute effect size of dietary intervention was limited. One mouse in the CR-SD group was found dead on day 22 form an undetermined cause. CR-KD in combination with MCP resulted in tumor regression all cases of SH-SY5Y and SK-N-BE(2). The fractional product of Webb [44] on the last day with available control tumor data showed synergism for KD and CR-KD for SH-SY5Y and CR-SD and KD for SK-N-BE(2). For the CR-SD an additive effect and for the CR-KD in SK-N-BE(2) antagonism was observed (Supplementary Table S3). These results need to be interpreted with caution due to the small tumor size in the SK-N-BE(2) and the lack of dosing curves [44].

### Effect of dietary intervention on blood glucose and ketone body levels

**Figure 2:**
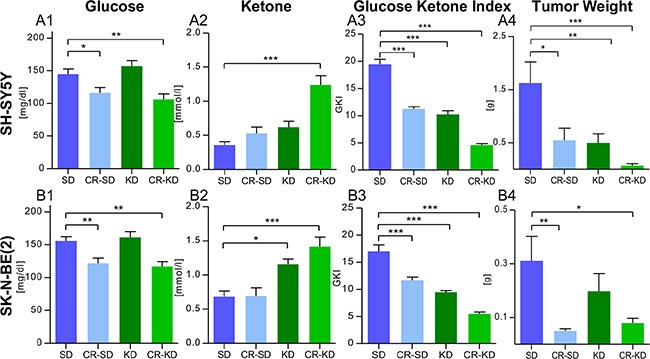
Blood glucose reduction and induction of ketosis goes with reduced tumor weight in mice under MCP (**2A**) SH-SY5Y groups and (**2B**) SK-N-BE(2) groups. (**A1**) and (**B1**) CR significantly reduced blood glucose levels in mice with both xenograft types. (**A2**) and (**B2**) Ketone body levels (beta-hydroxybutyrate) were consistently elevated in the CR-KD groups and the KD group of SK-N-BE(2). The trend in the KD group of SH-SY5Y did not reach statistical significance. (**A3**) and (**B3**) Mean Glucose Ketone Index over the treatment period was significantly reduced in all dietary intervention groups (*p* < 0.001). (**A4**) and (**B4**) Tumor weight was significantly reduced in all groups on MCP when compared to the SD group w/o CTx (*p* < 0.001; data not shown) and dietary intervention groups as given. The results are consistent with the tumor volumes calculated in Figure 1A and 1B. Data are shown for day 36 or the last day of therapy. Mean values ± SEM of the corresponding therapy group are given (*n* = 8–12). Statistics: ANOVA (*p* < 0.05) followed by two-tailed Dunnett's test correcting for multiple comparisons; *p ≤ 0.05; **p ≤ 0.01; ***p ≤ 0.001. Abbrev.: SD, standard diet; CR, calorie restriction; KD, ketogenic diet; w/o CTx, without chemotherapy; MCP, metronomic cyclophosphamide.

KD and/or CR caused consistent changes in blood glucose and ketone body levels in mice carrying the two different xenograft types. Data are given for day 36 or the last day of therapy (Figure 2A1–2A2 and 2B1–2B2). Blood glucose levels were significantly decreased in all four CR groups when compared to the SD group. In detail, blood glucose levels in SD group of SH-SY5Y were significantly higher when compared to CR-SD (*p* < 0.05) and CR-KD (*p* < 0.01). Ketone body levels were significantly elevated in the CR-KD group (*p* < 0.001) compared to the SD group, but not in the CR-SD. Glucose in the SK-N-BE(2) SD group was significantly higher compared to CR-SD (*p* < 0.01) and CR-KD (*p* < 0.01). Ketone body levels were significantly elevated in both the KD (*p* < 0.05) and CR-KD (*p* < 0.001) groups when compared to the SD group, but not in the CR-SD group. No significant changes in blood glucose were detected for neither of the KD groups. Detailed blood glucose and ketone body data for all time points is given in Supplementary Table S1. As published recently [45] the glucose ketone index (GKI), a ratio of blood glucose [mM] to blood ketone [mM] levels might help to monitor therapeutic efficacy in patients on ketogenic diets. Under the given food regimens, mice showed a GKI > 12 in the SD groups and GKI < 5 in the CR-KD diet group, on the last day of therapy. These ratios are consistent with other preclinical studies in mice [45]. Mean GKI is given in Figure 2A3 and 2B3 and shows a significant reduction in all dietary intervention groups (*p* < 0.001). GKI for all time points are given in Supplementary Table S2.

### Tumor and body weight

As tumor volume calculated by the described formula could be regarded as an approximation of tumor size, tumor weight was recorded after sacrificing mice. In line with the tumor volumes, tumor weight was significantly decreased in all groups on MCP (*p* < 0.001). Dietary intervention significantly enhanced suppression of tumor growth, evaluated as tumor weight, in all therapy groups (*p* < 0.05) except for the KD group of SK-N-BE(2) (Figure 2A3 and 2B3). No significant change in the tumor volume (mm^3) to weight (mg) ratio was observed (*p* > 0.05) (Supplementary Figure S1). Treatment with MCP did not significantly change mouse weight on the last day of therapy in mice on SD or KD (*p* > 0.05) (Supplementary Figure S2). The combination of MCP with CR caused a significant change in mouse weight for all four therapy groups when compared to the corresponding SD group (*p* < 0.05). For one mouse in the CR-KD group therapy was discontinued due to progressive weight loss on day 29. Detailed body weight data for all time points is given in Supplementary Table S1.

### Effect of MCP and dietary intervention on proliferation indices in NB xenografts

**Figure 3:**
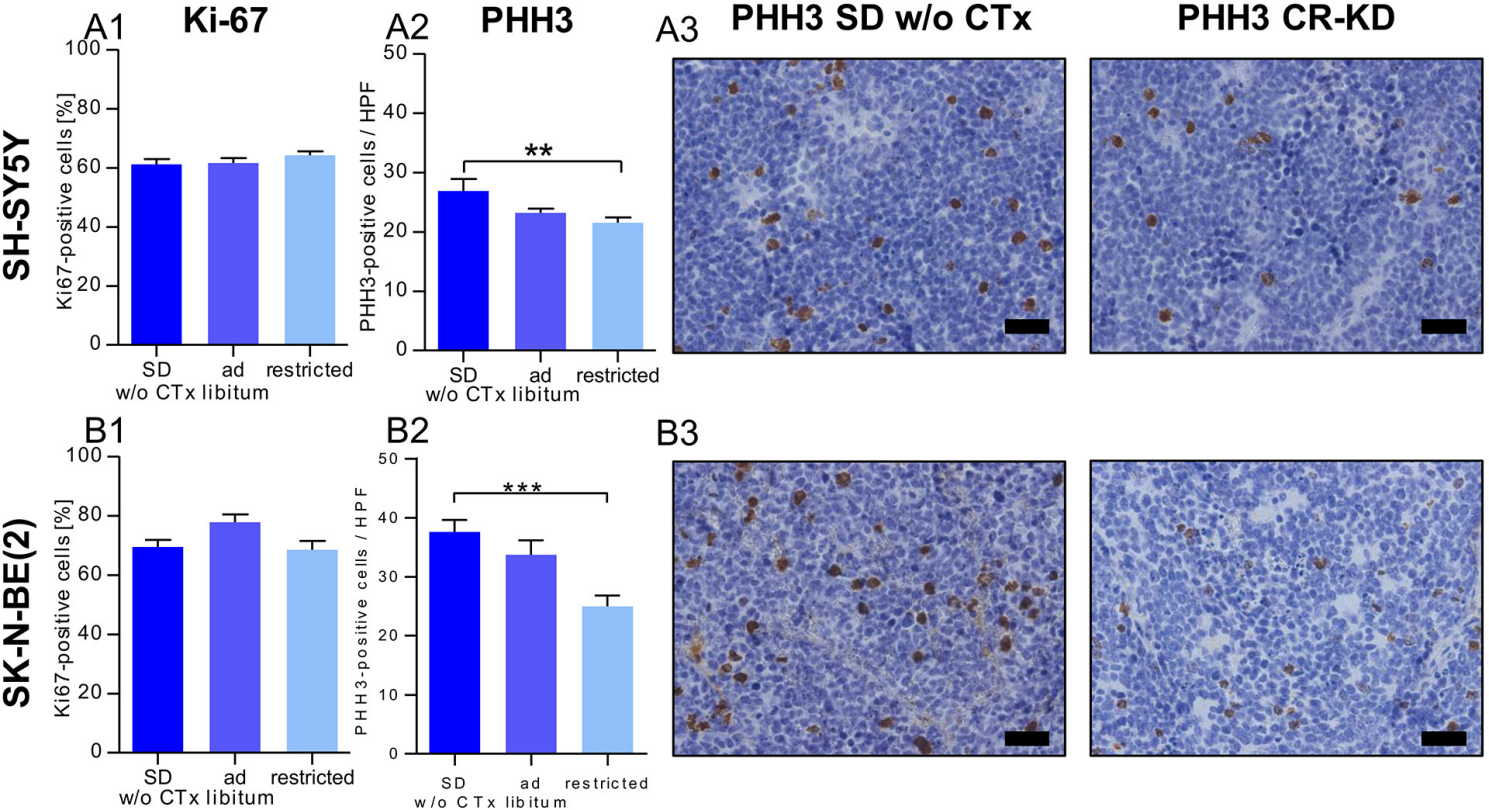
Reduced proliferation contributes to the growth inhibitory effect of CR but not MCP (**3A**) SH-SY5Y groups and (**3B**) SK-N-BE(2) groups. IHC evaluation of proliferation markers on day 36 or the last day of therapy. (**A1**) and (**B1**) The fraction of Ki67 positive stained cells was not significantly altered by MCP or diet when compared to SD w/o CTx. (**A2**) and (**B2**) In xenografts of both cell lines, PHH3 positive cells per high power field were significantly lower in the CR groups. (**A3**) and (**B3**) Exemplary tumor sections stained for PHH3 from mice on SD w/o CTx (left) and CR-KD (right) are shown. Scale bar = 50 μm. Mean values ± SEM of the therapy group are given (*n* ≥ 8). Statistics: ANOVA (*p* < 0.05) followed by two-tailed Dunnett's test correcting for multiple comparisons; **p ≤ 0.01; ***p ≤ 0.001. Abbrev.: Ad libitum: SD/KD; restricted: CR-SD/CR-KD. SD, standard diet; CR, calorie restriction; KD, ketogenic diet; w/o CTx, without chemotherapy. Proliferation indices for individual dietary subgroups are given in Supplementary Figure S3.

To further elucidate the tumor growth inhibitory effect of the combination of MCP and/or dietary intervention, we evaluated proliferative activity by scoring the markers Ki67 and PHH3. The staining patterns revealed similar effects of MCP and dietary intervention on proliferation indices in xenografts of the two cell lines. Ki67 levels did not differ significantly when comparing the non-restricted and CR groups receiving MCP treatment to the corresponding SD group w/o CTx (*p* > 0.05; Figure 3A1 and 3B1). The expression of PHH3, a marker for M-phase of the cell cycle, was significantly lower in the CR SH-SY5Y xenografts (*p* < 0.01, Figure 3A2) as well as in CR SK-N-BE(2) xenografts (*p* < 0.001, Figure 3B2). Proliferation indices for each individual subgroup SD/KD and CR-SD/CR-KD are given in Supplementary Figure S3. Together these results indicate that reduced cell division might play a role in the growth inhibitory effect of CR, but does not explain the pronounced effect of MCP in the more chemotherapy-resistant (SK-N-BE(2)) xenografts.

### *In vitro* 4-hydroperoxycyclophosphamide (4-HC) sensitivity

Because the *in vivo* results showed MCP to be more active against xenografts of the reportedly chemotherapy-resistant cell line SK-N-BE(2), we compared the *in vitro* CP-sensitivity of SH-SY5Y and SK-N-BE(2) to confirm the data reported in the literature [41–43]. We therefore titrated 4-HC concentration (active metabolite of CP) to a dose-range, in which both cell lines showed either complete cell death (10 nM) or no difference in survival (0.313 nM) under the conditions tested. At all intermediate doses SH-SY5Y showed significantly higher sensitivity to 4-HC (Supplementary Figure S6). From these data we hypothesized that, rather than a direct cytotoxic effect, an indirect effect on xenograft growth may be rendering SK-N-BE(2) tumors more susceptible to MCP. To our knowledge this has not been shown in literature before.

### Macroscopic observation of intratumoral hemorrhage

**Figure 4:**
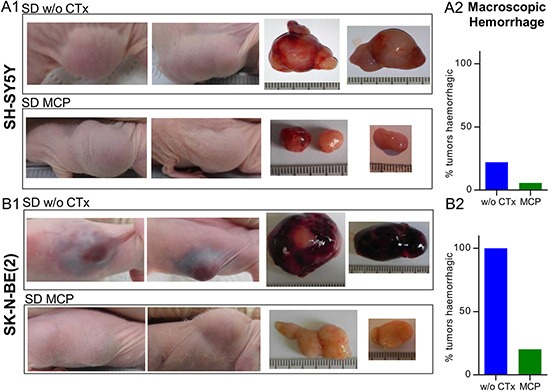
Predominant intratumoral hemorrhage in *MYCN*-amplified tumors is reduced upon MCP treatment (**4A**) SH-SY5Y and (**4B**) SK-N-BE(2) tumors. Images of xenograft sites, as well as tumors after explantation are shown. Pronounced macroscopic hemorrhage in the SK-N-BE(2) SD group w/o CTx compared to all other therapy groups of both cell lines was evident. (**A1**) and (**B1**) Specimens from SD w/o CTx compared to SD on MCP are shown. (**A2**) and (**B2**) Relative quantification of macroscopically visible hemorrhagic tumors w/o CTx (SH-SY5Y *n* = 9, SK-N-BE(2) *n* = 8) and groups on MCP (SH-SY5Y *n* = 35, SK-N-BE(2) *n* = 25). Intratumoral hemorrhage did not vary between dietary subgroups and is given in Supplementary Figure S4. Abbrev.: SD, standard diet; w/o CTx, without chemotherapy; MCP, metronomic cyclophosphamide.

Macroscopic inspection and evaluation of photo documentation showed a tendency to stronger intratumoral hemorrhage in the SK-N-BE(2) xenografts compared to SH-SY5Y xenografts (Figure 4A1 and 4B1). We therefore quantified macroscopic hemorrhage at day 36 or the last day of therapy (Figure 4A2 and 4B2). In the SK-N-BE(2) group on SD w/o CTx, 100% (8/8) of tumors showed pronounced macroscopic hemorrhage. On therapy with MCP this was reduced to 20% (5/25), which approximates the level of hemorrhage observed in SH-SY5Y xenografts (22%, 2/9) without MCP. In the SH-SY5Y xenografts, MCP reduced macroscopic hemorrhage to a level of 6% (2/35). Intratumoral hemorrhage did not vary between dietary subgroups and is given in Supplementary Figure S4.

### Effect of MCP on microscopic tumor vascularization, hemorrhage and vessel maturation

**Figure 5:**
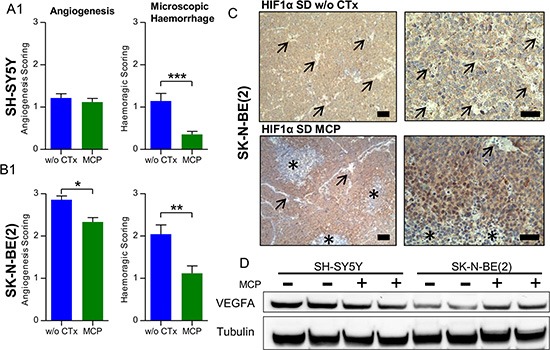
Microscopic evaluation supports an anti-angiogenic effect of MCP and vessel maturation in NB xenografts (**5A1**) SH-SY5Y and (**5B1**) SK-N-BE(2). Hematoxylin/Eosin staining of NB sections on day 36 or the last day of therapy was scored for angiogenesis and hemorrhage on a scale from 0-3. In the SK-N-BE(2) xenografts, blood vessel morphology changed to a more mature pattern as described in detail in Figure 6B1 and 6B2. (**C**) IHC staining for HIF1A of a SK-N-BE(2) xenograft section from the SD group w/o CTx (upper) and SD on MCP (lower). Blood vessel (arrow) rarefication and maturation caused a hypoxic pattern in SK-N-BE(2) xenografts with increased nuclear HIF1A accumulation and areas of marked cell death in the center of hypoxic areas (asterisk). (**D**) This correlated to VEGFA up regulation in SK-N-BE(2) xenografts exposed to MCP, as depicted by immunoblotting. Scale bars = 100 μm (overview) and 50 μm (detail). Mean values ± SEM of the therapy group are given (*n* ≥ 8). Statistics: unpaired *t* test (*p* < 0.05); *p ≤ 0.05; **p ≤ 0.01; ***p ≤ 0.001. Angiogenesis scoring across all subgroups is given in Supplementary Figure S5. Densitometry for VEGFA levels is given in Supplementary Figure S8A. Abbrev.: SD, standard diet; w/o CTx, without chemotherapy; MCP, metronomic cyclophosphamide.

**Figure 6:**
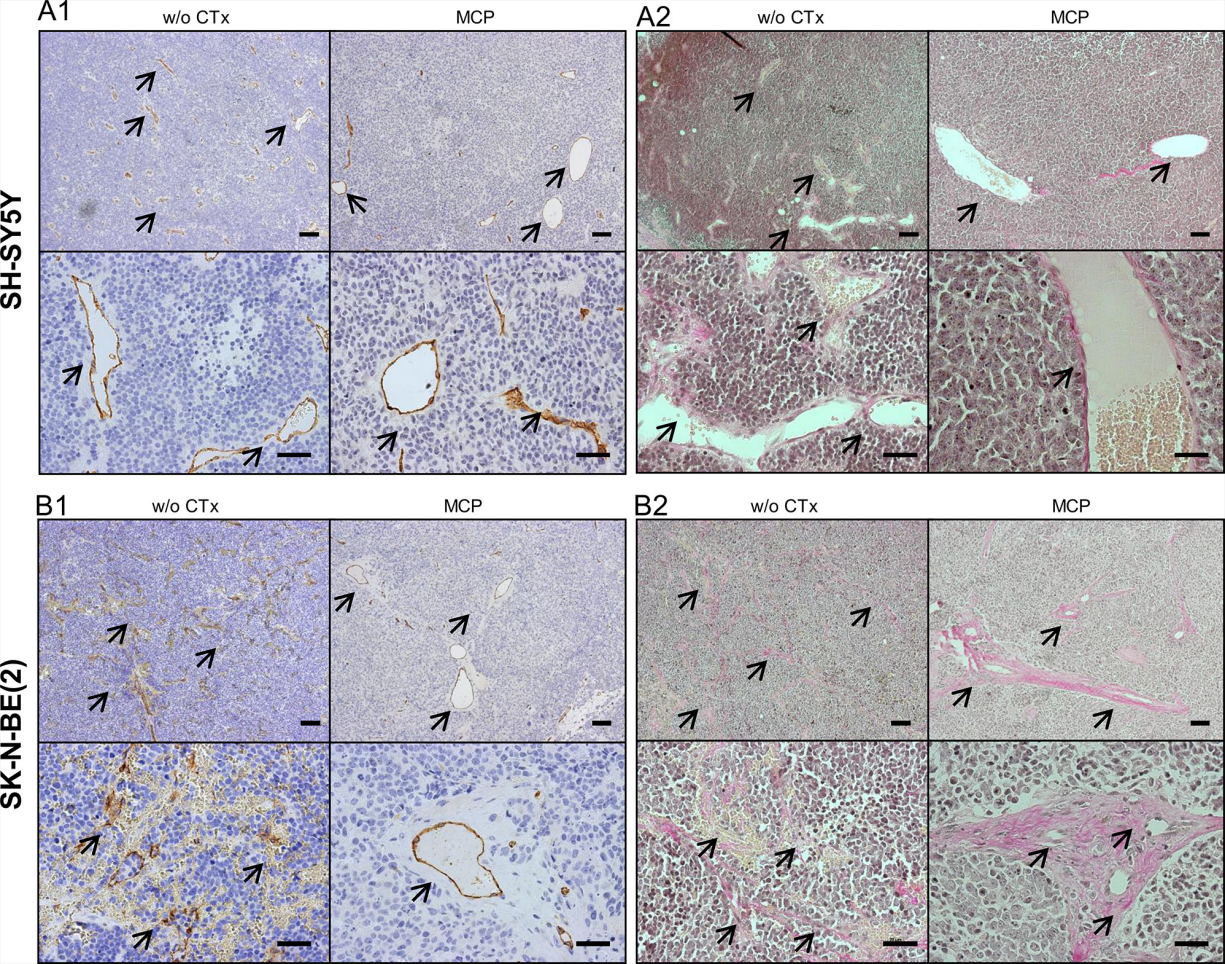
Evaluation of blood vessels by IHC for msCD-31 endothelial cell marker (A1, B1) and Van Gieson staining of collagen fibers (A2, B2) Images show exemplary sections of (**6A**) SH-SY5Y and (**6B**) SK-N-BE(2) xenografts at day 36 or the last day of therapy. The left upper (overview) and lower (detail) images are taken from tumors w/o CTx, the right upper and lower images are from tumors exposed to MCP. (**A1**) and (**B1**) show IHC staining for the endothelial marker msCD-31. Under both conditions SH-SY5Y xenografts show well-formed blood vessels, as can be appreciated best in the detail section (A1, lower). In comparison SK-N-BE(2) xenografts show marked difference in blood vessel morphology between the w/o CTx group and the MCP group (B1). (A2) and (B2) Van Gieson staining identified the strong perivascular connective tissue in the SK-N-BE(2) group on MCP as collagen deposition (red). Blood vessels are marked by arrows, Scale bars = 100 μm (overview) and 50 μm (detail). Abbrev.: SD, standard diet; w/o CTx, without chemotherapy; MCP, metronomic cyclophosphamide.

To further substantiate the results from macroscopic inspection, tumor vascularization and intratumoral hemorrhage were studied on tumor sections stained with Hematoxylin/Eosin (Figure 5A1 and 5B1). In line with the macroscopic observations, vascularization and hemorrhage were significantly higher in the SK-N-BE(2) xenografts compared to SH-SY5Y tumors. Upon MCP treatment, hemorrhage in both xenograft types was significantly reduced (*p* < 0.01). Along with the significant reduction of vascularization in the SK-N-BE(2) xenografts (*p* < 0.05), maturation of blood vessels was observed in xenografts of this cell type (Figure 6B). Angiogenesis scoring across all subgroups is given in Supplementary Figure S5. The change in vessel structure seemed even more fundamental than the reduction of vessel number. Increased nuclear accumulation of HIF1A was evident in the SK-N-BE(2) xenografts exposed to MCP. This was accompanied by central regions of cell death (Figure 5C) and VEGF up-regulation (Figure 5D). Increased VEGFA could not compensate for the anti-angiogenic effect of the MCP therapy. It can be speculated that the marked increase in diffusion distance (as can be seen in Figure 6B1 and 6B2, right) likely contributes to the increasing tissue hypoxia and cell death as depicted in Figure 5C.

Endothelial cell marker msCD-31 and Van Gieson staining were used to further characterize the nature of the prominent hemorrhage in SK-N-BE(2) xenografts. msCD-31 staining of SK-N-BE(2) tumors showed a poorly defined vascular pattern largely lacking both mature vessel structure and a basal membrane. Blood vessels of all diameters were strongly invaded by NB tumor cells, resulting in erythrocyte leakage into the tumor (Figure 6B1 and 6B2, left). Only rare blood vessels in the periphery of SK-N-BE(2) xenografts showed an intact vessel structure with prominent basal lamina, as was consistently observed in the SH-SY5Y xenografts (Figure 6A1 and 6A2 left). Under MCP treatment, however, in addition to a reduction of overall blood vessel density, a pronounced maturation of blood vessels was evident in SK-N-BE(2) tumors (Figure 6B1 and 6B2, right). No tumor cell invasion into vascular structures was observed in SK-N-BE(2) xenografts exposed to MCP. This change of pattern was not observed in SH-SY5Y xenografts, where both treatment groups showed relatively-well developed vascular patterns (Figure 6A1 and 6A2). Compared to SD tumors w/o CTx MCP treated tumors showed a tendency towards decreased cellularity and reduced nucleus/cytosol ratio in both SH-SY5Y and SK-N-BE(2) xenografts.

### Diametrically opposed regulation of Bcl-2 protein levels in SH-SY5Y and SK-N-BE(2) tumors under MCP

**Figure 7:**
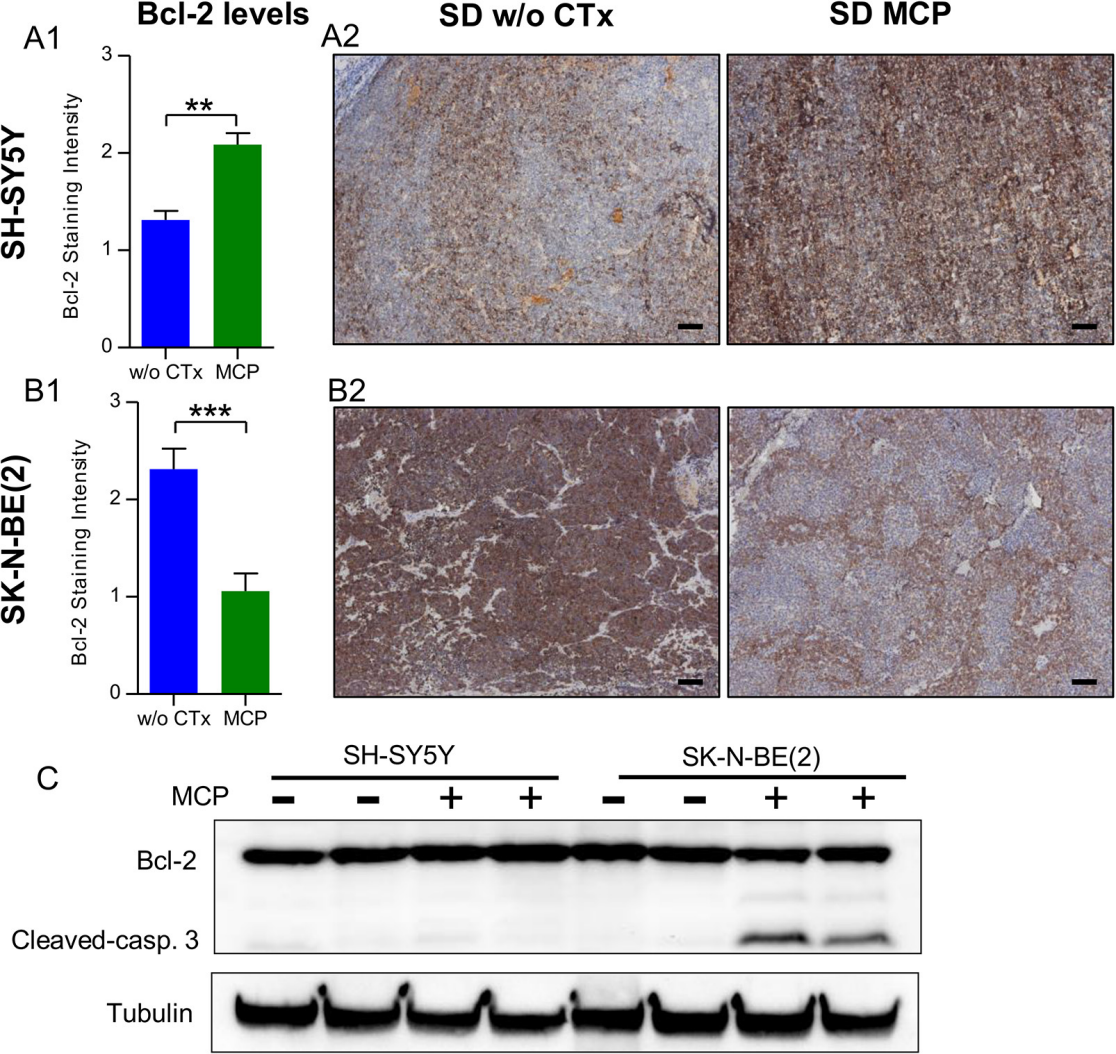
SH-SY5Y (A) and SK-N-BE(2) (B) cell lines show divergent regulation of Bcl-2 levels under MCP (**A1**) and (**B1**) Bcl-2 levels on IHC staining were scored on day 36 or the last day of therapy. In the untreated state, SH-SY5Y xenografts showed significantly lower Bcl-2 levels when compared to SK-N-BE(2) xenografts (*p* < 0.001). On MCP treatment, the two cell lines showed an opposing response. (**A2**) and (**B2**) Exemplary Bcl-2 IHC-stained sections w/o CTx (left) and on MCP (right). (**C**) On western blot analysis, along with the reduction in Bcl-2 levels, increased cleaved caspase 3 protein was detected in the SK-N-BE(2) xenografts exposed to MCP. Densitometry for western blots is given in Supplementary Figure S8B. Scale bar = 100 μm; Mean values ± SEM of the therapy group are given (*n* ≥ 8). Statistics: Stundent's *t* test (*p* < 0.05); **p ≤ 0.01, ***p ≤ 0.001. Abbrev.: SD, standard diet; w/o CTx, without chemotherapy; MCP, metronomic cyclophosphamide.

For both cell lines used in our study, Bcl-2 expression was described previously [46] and was confirmed by IHC-staining in our study. Under continuous MCP treatment, SH-SY5Y xenografts of mice on SD showed a progressive increase in tumor volumes starting around treatment day 16. Along with this resistance, significantly higher Bcl-2 (*p* < 0.01; Figure 7A) expression was evident in xenografts exposed to MCP treatment compared to SD w/o CTx. This is in line with the literature for NB tumors, which suggest Bcl-2 upregulation as a mechanism of chemotherapy resistance under standard treatment [47, 48]. Consistent with the tumor cell characteristics [48] (chemotherapy resistant and *MYCN* amplified) the SK-N-BE(2) cell line showed increased basal Bcl-2 protein expression levels in the untreated group when compared to SH-SY5Y. On MCP treatment, however, Bcl-2 protein levels were significantly reduced in the SK-N-BE(2) xenografts (*p* < 0.001; Figure 7B). Whereas Bcl-2 staining was more preserved in vascularized areas, it was particularly low with increased distance to blood vessels (Figure 7B2, right). Western blot analysis (Figure 7C) supports the results from IHC. Whereas SH-SY5Y tumors exhibited slightly increased Bcl-2 levels upon prolonged MCP treatment, SK-N-BE(2) tumors showed decreased levels. Concomitant with the decrease in Bcl-2 levels, an increase in cleaved caspase 3 was observed in MCP-treated SK-N-BE(2) xenografts. These results support the higher sensitivity of SK-N-BE(2) tumors to MCP as observed by tumor volume.

## DISCUSSION

The present study combines two alternative therapeutic approaches to reduce NB tumor growth in a preclinical model. In this setting, administering an anti-angiogenic MCP regimen with dietary intervention was able to significantly inhibit growth of NB xenografts of distinct genetic background and chemotherapy sensitivity.

In line with our previous work that focused exclusively on the effect of dietary intervention [21], KD and/or CR reduced glucose availability and increased blood ketone body levels in mice. Significant reductions of tumor growth, in addition to MCP treatment, was evident in all dietary intervention groups except for the SK-N-BE(2) xenograft group on KD. Although dietary effects in the SK-N-BE(2) subgroup should be interpreted with caution due to the small absolute tumor size, results of different subgroups align with our previous work [21]. This supports the metabolic dependency of NBs and their limited capacity to adapt to the change in nutrient supply.

In contrast to other studies on the effect of KD and/or CR on tumor growth [21, 49–51], we not only observed growth inhibition, but also regression of tumor volume. Intriguingly, the combination of MCP and CR-KD induced a significant reduction of tumor size and complete growth arrest of NB xenografts of both cell lines tested. This highlights the importance of combining dietary intervention with other therapeutic approaches as it might sensitize tumors to the cytotoxic effects of these therapeutics. In line with our data, enhancement of anti-tumor effect has been described in additional preclinical studies [52–54].

For NB, increased vascularization has been proposed to correlate with high-risk tumor stage and poor clinical outcome [55–57]. Targeting neovascularization has therefore been put forward as a therapeutic option [58–61]. Furthermore, a direct link between *MYCN* amplification and enhanced angiogenesis has been described [55, 62, 63]. In accord we observed increased angiogenesis and a highly invasive growth pattern in xenografts of the *MYCN-*amplified cell line. Upon MCP treatment, this phenotype changed to a mature vessel pattern with strong perivascular collagen deposition and without vascular invasion of tumor cells. To our knowledge this effect has not been described before. The change in vessel structure presumably resulted in undersupplied tumors due to the increased diffusion distance. This interpretation is supported by the increase in hypoxic areas with central cell death as seen on HIF1A staining. Direct evidence for nutrient deprivation, however, is pending.

Whereas the SH-SY5Y xenografts in the SD group developed resistance to the MCP regimen that was accompanied by up-regulation of Bcl-2 levels, in the reportedly more chemotherapy-resistant SK-N-BE(2) xenografts [41–43], sensitivity was retained. The increased *in vitro* chemotherapy resistance of the SK-N-BE(2) cells supports an anti-tumor mechanism of MCP that is not linked to a direct cytotoxic effect. Previous reports showed that *in vitro* hypoxia can trigger an autocrine loop via increased VEGF expression to induce Bcl-2 mediated chemotherapy resistance in SK-N-BE(2) cells [64, 65]. Although our model supports findings of increased VEGFA expression in response to hypoxia, this loop was not sufficient to induce Bcl-2-mediated tumor cell survival in the *in vivo* setting.

This finding might highlight the importance of a concomitant decrease in nutrient supply that accompanies the state of low vascularization in tumors which is often not prioritized in *in vitro* studies [64, 65]. Supporting published work on an anti-angiogenic effect of KD [66], we observed the strongest reduction of angiogenesis in the combination of KD with MCP. Whereas both publications report a rarefication of blood vessels and hemorrhage, the fibrous depositions and vessel maturation are only described in our model.

Although we are aware of the limitations of an ectopic xenograft model and potential effects of additional molecular differences between the cell lines, the pronounced effect on tumor growth inhibition and blood vessel maturation could help to further clarify mechanisms in this molecular subgroup of NBs [62]. It can be hypothesized, that along with the increased vascularization, also a stronger dependency on vascular supply is present for promoting tumor growth. Together with targeting cell metabolism this might constitute an Achilles heel worth aiming for to improve treatment for therapy-refractory NB.

## MATERIALS AND METHODS

### Cell lines

Neuroblastoma xenografts were established with the cell lines (ATCC, Manassas, VA, USA) SH-SY5Y (CRL-2266) and SK-N-BE(2) (CRL-2271). SH-SY5Y is a *TP53* wild type, non-*MYCN-*amplified cell line with no chromosome 1p loss of heterozygosity and is sensitive to chemotherapy. The SK-N-BE(2) cell line is highly resistant to a wide range of chemotherapeutic agents and is characterized by *MYCN*-amplification, *TP53* mutation (p.C135F) and chromosome 1p loss of heterozygosity [41]. Cells were cultured in standard conditions as described earlier [21]. MYCN expression on protein level was confirmed on western blot and is given in Supplementary Figure S7.

### Animal models and sample preparation

All *in vivo* experiments were performed in accordance with protocols approved for this study by the Salzburg Animal Care and Use Committee (Nr.: 20901-TVG/44/7-2011). This protocol specifically addressed weight loss observed in response to dietary intervention. Animals were maintained under specific pathogen-free conditions and care conformed to the Austrian Act on Animal Experimentation. Xenograft growth was induced on the right flanks of 5-to 6-week-old female CD-1 nude mice (Charles River, Wilmington, MA, USA) by subcutaneous injection of a 200 μl suspension of NB cells (2.7 × 10^7^) in serum-free medium and matrigel (BD Bioscience, Franklin Lakes, NJ, USA). After reaching a tumor size of 350 mm^3^, an oral metronomic cyclophosphamide treatment was started and mice were randomized to four dietary therapy groups (SD, CR-SD, KD and CR-KD; *n* = 10–12). One control group on standard diet without chemotherapy was included for both cell lines (SD w/o CTx; *n* = 8–9).

Two times per week, tumor volumes were measured using a caliper and calculated according to the formula width × height × length/2. Body weight was recorded and blood glucose and ketone body (beta-hydroxybutyrate) levels were monitored using enzyme based methods twice weekly (Precision Xceed, Abbott Laboratories, North Chicago, IL). In the ad libitum-fed groups, measurements were performed after a two-hour fasting period or before feeding in the calorie restricted groups. Animals in CR groups were single-housed to control food intake. Ad libitum-fed mice were group housed. Mice were sacrificed at day 36 or when termination criteria were met (health status, tumor ulceration or volume of 3000 mm^3^). Images of the xenografts were recorded at the start of therapy, and before and after removal of the tumor. Cancer tissue was snap frozen in liquid nitrogen. One 0.5 cm tumor slice each was formalin-fixed and paraffin embedded for histological analysis.

### Food composition and energy content

Mice were fed according to four different regimens: standard diet ad libitum (SD), calorie restricted standard diet (CR-SD), long-chain fatty acid-based ketogenic diet ad libitum (KD) and calorie-restricted ketogenic diet (CR-KD). Detailed food composition and feeding protocols to evaluate the dietary intervention on NB-tumor growth were published previously [21] and are given in the Supplementary Data (Table S4). In brief, the metabolizable energy contents were: SD (kcal: fat 9%, protein 33% and carbohydrates 58%) and mild KD (kcal: fat 78%, protein 14% and carbohydrates 8%) (No. V1535-000 and No. S9139-E02D; Sniff Spezialdiäten GmbH, Soest, D). Diets were fortified with vitamins and mineral supplements. For the CR groups, food intake was restricted to 2/3 of the respective ad libitum intake, which aligns with clinical protocols for therapy-resistant epilepsy [67–69].

### Metronomic cyclophosphamide (MCP) schedule

CP (Sigma, St Louis, MO, USA) was administered orally through the drinking water as described previously [70]. In order to achieve the described oral dose of 40 mg/kg CP per day, final CP concentration in drinking water was 0.266 mg/ml [70]. Analogous MCP regimens were applied to target neoangiogenesis in xenograft models of other tumor entities [34–36] and have low toxicities [70, 71]. Average water/CP intake per mouse was equal between different diet groups.

### *In vitro* CP sensitivity

*In vitro* CP sensitivity of both cell lines was evaluated by utilizing 4-hydroperoxycyclophosphamide (4-HC, Niomech, Bielefeld, D), an active metabolite of CP. Cells were seeded in 96 well plates (25 000/well). After resting for 48 hours, standard medium was replaced by 100 μl medium containing 4-HC (0.157 – 10 nM). 4-HC was freshly dissolved in DMSO (Sigma), and control medium contained the maximum DMSO concentration (0.01%). After three hours of drug exposure, the medium was replaced by 100 μl standard medium for 24 hours to allow the cytotoxicity to fully develop. 3-(4,5-dimethylthiazol-2-yl)-2, 5-diphenyltetrazolium bromide (MTT) assay was performed following the manufacturer's instructions (Life Technologies, Carlsbad, CA, USA). Absorbance was read at 570 nm with an EnSpire Multimode Plate Reader (PerkinElmer, Waltham, MA, USA). Results were normalized to background and are given as metabolic activity relative to control (100%). The average of two independent experiments with 6 replicates each is shown.

### (Immuno-)histochemical staining and analysis

Histological staining was performed using 5-μm deparaffinized tumor sections of NB xenografts. Hematoxylin/eosin and Van Gieson staining as well as immunohistochemistry (IHC) stainings (Bcl-2 (B-cell lymphoma 2), PHH3 (phospho-histone H3), Ki67) were carried out within the routine diagnostic setting at the local pathology department following standard protocols. Antibodies were used at the following dilutions: 1:100 for anti-Bcl-2 (Dako, Glostrup, Denmark), 1:200 for anti-PHH3 (Cellmarque, Rocklin, CA, USA) and 1:500 for anti-Ki67 (Dako). Angiogenesis, intratumoral hemorrhage and Bcl-2 staining were scored on a scale of 0 (none) to 3 (strong). Proliferation indices were scored by evaluating the proportion of positively stained nuclei scoring at least 500 cells per slide (Ki67) or counting the number of positively stained tumor nuclei per high-power field (PHH3/HPF). For adequately sized tumors, at least five representative regions were scored; magnification was 400-fold. When the tumor size was too small, stained nuclei/total tumor cells were counted and converted to a HPF equivalent. Anti-msCD31 (Abcam, Cambridge, UK) and anti-HIF1A (hypoxia-inducible factor 1-alpha, Novus, Littleton, USA) stainings were carried out at a dilution of 1:50 and 1:100, respectively, following protocols for IHC-staining described earlier [19, 72]. For detection EnVision kit reagents (Dako) were used following the manufacturer's instructions. Analysis of histological staining was performed by two blinded investigators, and the mean of both scores was calculated.

### Western blot analysis

Whole-cell lysates for western blot analysis were prepared using standard radioimmunoprecipitation assay buffer. In brief, 30 μg protein were separated on 10% acrylamide/bis-acrylamide gels before transfer to polyvinylidene difluoride membranes (Bio-Rad, Hercules, CA, USA) using CAPS buffer (10 mM 3-[cyclohexylamino]-1-propane sulfonic acid, pH 11; 10% methanol). The following primary antibodies were diluted in 5% w/v BSA (Sigma) in TBS-T: 1:1000 anti-Bcl-2 (Dako), 1:1000 anti-cleaved caspase 3 (Cell Signaling Technologies, Danvers, MA, USA), 1:30 000 anti-tubulin (Promega, Madison, WI, USA), 1:1000 anti-VEGFA (vascular endothelial growth factor, Abcam) and 1:1000 anti-NMYC (Abcam). Horseradish peroxidase-labeled secondary antibodies were used (Dako) and detection was carried out with Lumi-Light POD-substrate (Roche, Basel, Switzerland). Quantification of band intensities was performed using Image Lab Software 5.2.1 (Bio-Rad) and adapted to the corresponding loading control.

### Statistics

Statistical analysis was performed by Student's *t* test (two groups; *p* < 0.05) and one-way ANOVA (more than two groups; *p* < 0.05). To adapt for multiple comparisons two-tailed Dunnett's posttest was used. In these cases multiplicity adjusted *p*-value is given. If not mentioned otherwise, results are given as mean ± SEM. The analyses were performed using Prism 6 (GraphPad Software, La Jolla, CA, USA) and SPSS 21 (IBM, Armonk, NY, USA). The fractional product of Webb was used to determine whether the observed effects show synergistic or additive nature. The method uses the formula: i_1,2_ = i_1_*i_2_; where i_1,2_ is the inhibitory fraction of the combination of two interventions i_1_ (cyclophosphamide) and i_2_ (diet). When the predicted response exceeds the measured response, synergism is claimed (Ratio calculated/hypothetical < 1) [44].

## SUPPLEMENTARY MATERIALS FIGURES AND TABLES



## Abbreviations

NB: neuroblastoma
SD: standard diet
CR: calorie restriction
KD: ketogenic diet
w/o CTx: without chemotherapy
MCP: metronomic cyclophosphamide
