# Supplementary material for: Combination of metronomic cyclophosphamide and dietary intervention inhibits neuroblastoma growth in a CD1-nu mouse model
Source: Oncotarget. 2016 Mar 5;7(13):17060–73. doi: 10.18632/oncotarget.7929 (PMC4941371; doi:10.18632/oncotarget.7929)
Supplement: Supplementary file 1 [file oncotarget-07-17060-s001.pdf]

Combination of metronomic cyclophosphamide and dietary intervention inhibits neuroblastoma growth in a CD1-nu mouse model

Supplementary Materials

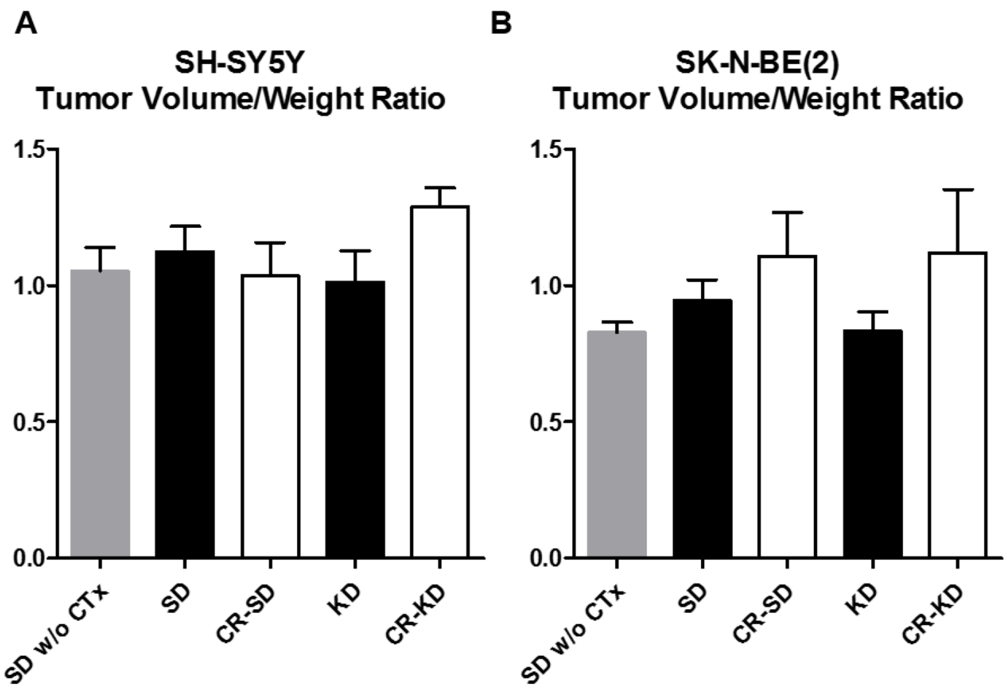

Supplementary Figure S1: Tumor volume (mm^3) to weight (mg) ratio.

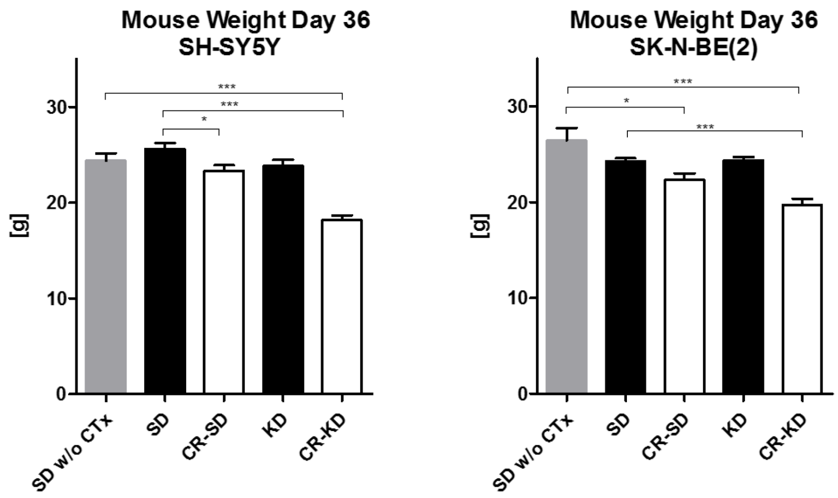

Supplementary Figure S2: Comparison of mouse weights on Day 36 or last day of therapy.

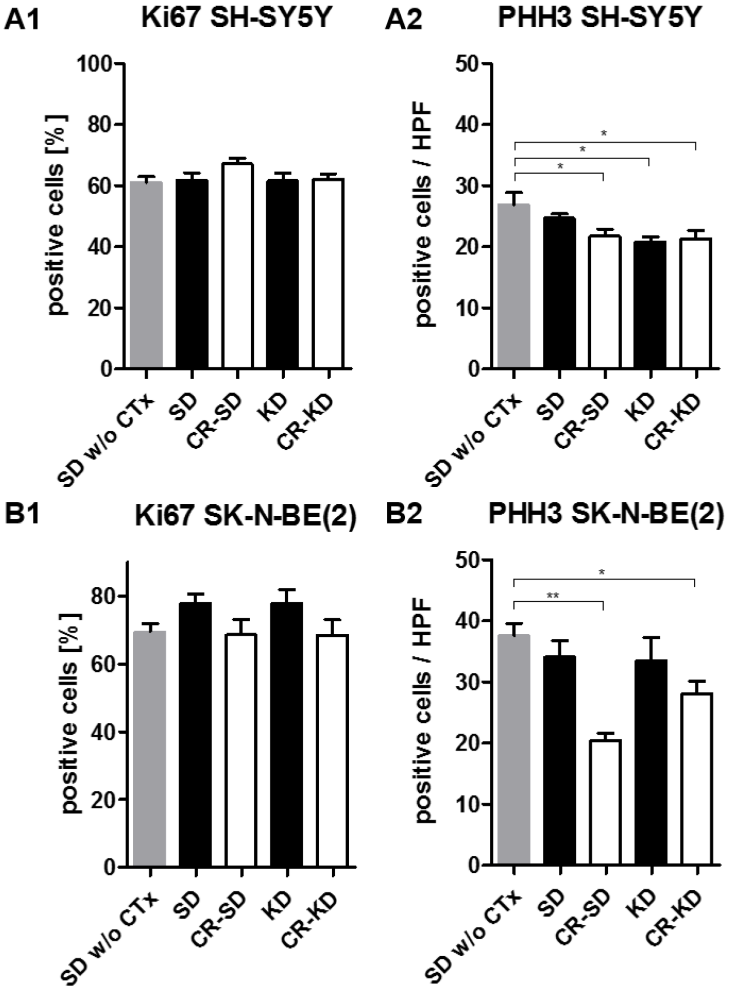

Supplementary Figure S3: Proliferation indices of dietary intervention subgroups.

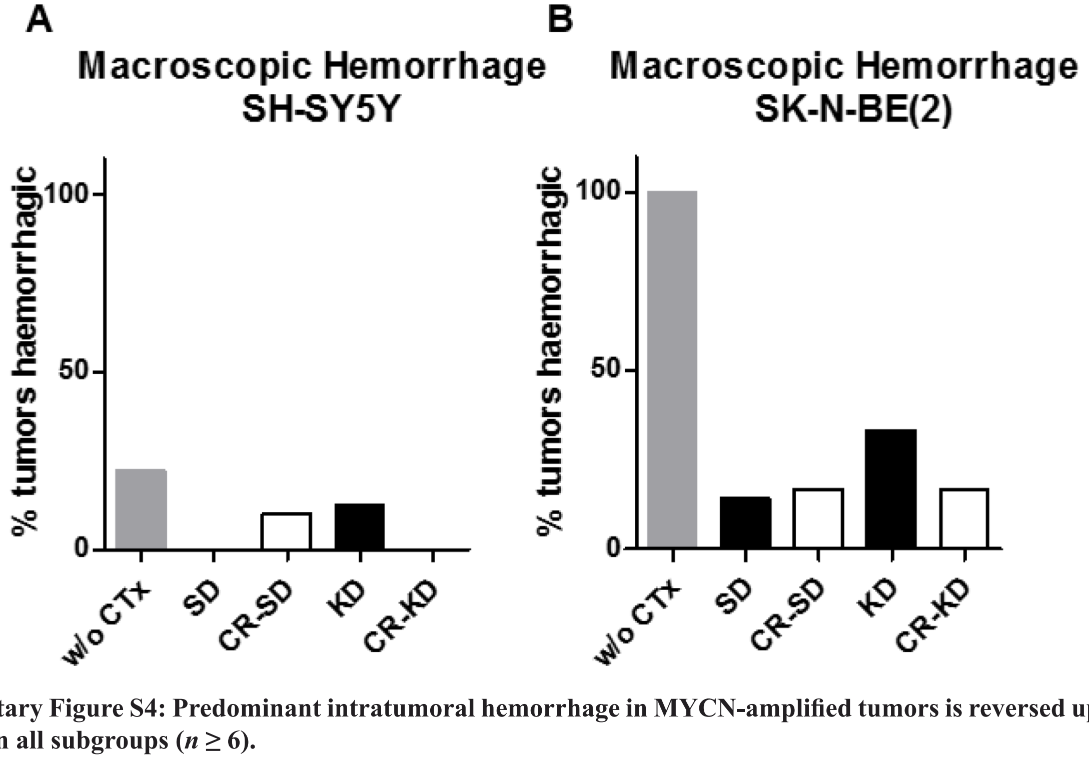

Supplementary Figure S4: Predominant intratumoral hemorrhage in MYCN-amplified tumors is reversed upon MCP treatment in all subgroups (*n* ≥ 6).

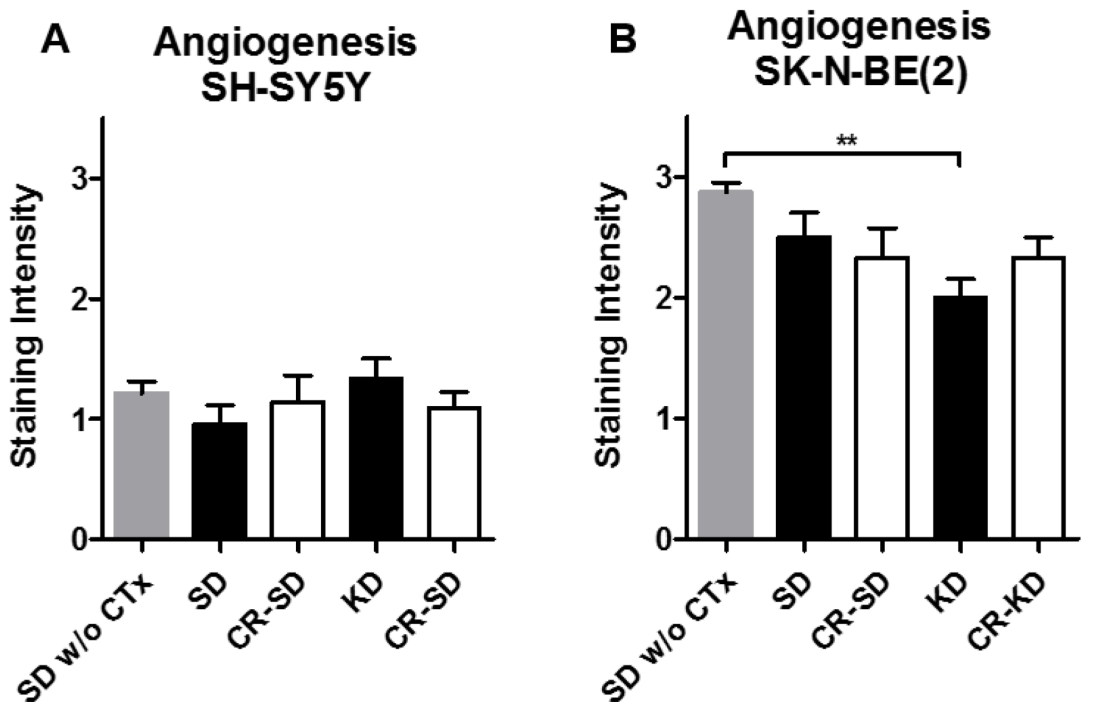

Supplementary Figure S5: Inhibition of angiogenesis by MCP and dietary intervention.

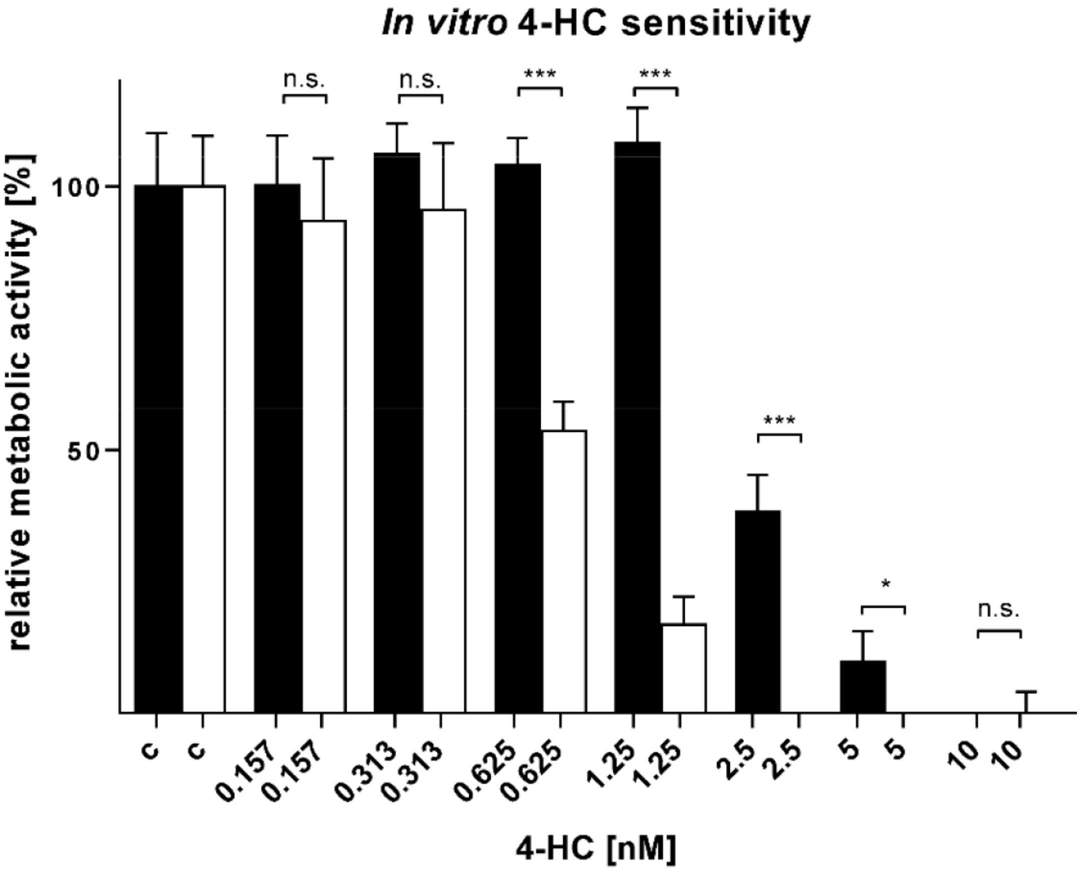

Supplementary Figure S6: *In vitro* 4-HC sensitivity. In line with the data reported in literature, the SH-SY5Ycell line (white bars) proofed significantly more susceptible to 4-HC than SK-N-BE(2) cells (black bars). Data is presented as mean ± SEM metabolic activity (MTT assay) of the corresponding 4-HC and normalized to control concentration. An average of two experiments is displayed (n = 12). Statistics: Student´s t test ($p < 0.05$); *$p \leq 0.05$; **$p \leq 0.01$; ***$p \leq 0.001$. Abbreviations: 4-HC 4-hydroperoxycyclophosphamide; MTT 3-(4, 5-dimethylthiazol-2-yl)-2, 5-diphenyltetrazolium bromide.

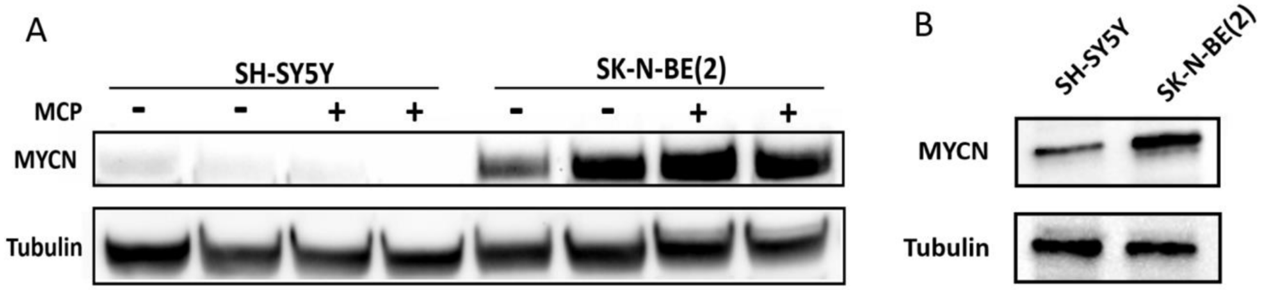

Supplementary Figure S7: Western blot of MYCN levels in SH-SY5Y and SK-N-BE(2). (**A**) xenografts and (**B**) whole cell lysates from tissue culture.

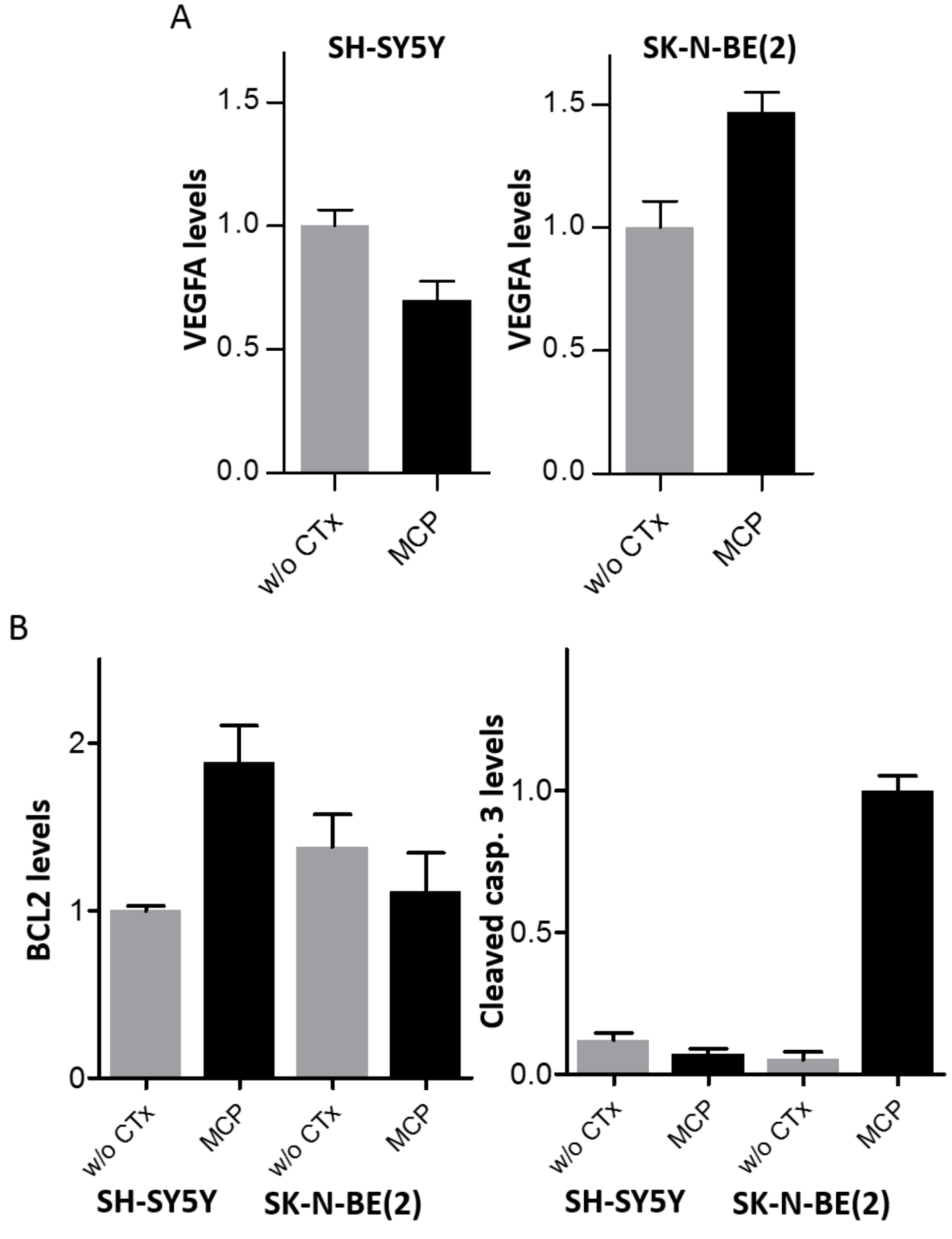

Supplementary Figure S8: Quantification of protein levels using Image Lab 5.2.1 software for (A) VEGFA western blot from Figure 5D and (B) BCL2 and Cleaved caspase 3 western blot from Figure 7C. Relative levels are shown with signal intensities normalized to the respective loading control (Tubulin).

Supplementary Table S1: Metabolic parameters blood glucose (BG), ketone body (KB) levels and body weight (BW)

A-SH-SY5Y

**Diet**	**SD w/o CTx**			**SD**			**CR-SD**			**KD**			**CR-KD**		
**day**	BG	KB	BW	BG	KB	BW	BG	KB	BW	BG	KB	BW	BG	KB	BW
**5**	148 ± 10	0.36 ± 0.05	24.8 ± 0.6	139 ± 6	0.31 ± 0.04	25.7 ± 0.6	113 ± 10	0.55 ± 0.07	25 ± 0.4	120 ± 10	0.84 ± 0.1	24.9 ± 0.5	94 ± 8	1.46 ± 0.24	25.7 ± 0.4
**8**	155 ± 8	0.34 ± 0.05	25.1 ± 0.8	148 ± 7	0.41 ± 0.04	25.1 ± 0.6	102 ± 6	0.56 ± 0.13	22.4 ± 0.6	142 ± 13	0.77 ± 0.05	23.2 ± 0.6	84 ± 5	1.23 ± 0.2	20.3 ± 0.5
**12**	149 ± 7	0.43 ± 0.08	25.1 ± 0.8	155 ± 7	0.47 ± 0.06	24.7 ± 0.5	112 ± 10	0.74 ± 0.11	22.2 ± 0.4	161 ± 9	0.79 ± 0.09	22.2 ± 1	100 ± 9	1.42 ± 0.2	18.7 ± 0.6
**15**	168 ± 12	0.45 ± 0.08	26.2 ± 0.7	156 ± 9	0.47 ± 0.08	25.7 ± 0.4	127 ± 9	0.62 ± 0.11	21.3 ± 0.4	162 ± 9	0.92 ± 0.09	22.8 ± 0.8	106 ± 5	1.45 ± 0.29	17.4 ± 0.6
**19**	148 ± 20	0.4 ± 0.14	25.8 ± 0.7	165 ± 7	0.43 ± 0.04	25.5 ± 0.5	131 ± 6	0.65 ± 0.07	21.6 ± 0.5	164 ± 8	0.96 ± 0.08	23.2 ± 0.8	117 ± 6	1.26 ± 0.24	16.9 ± 0.5
**22**				148 ± 7	0.52 ± 0.07	26 ± 0.5	126 ± 6	0.61 ± 0.09	21.4 ± 0.5	157 ± 6	1.11 ± 0.11	23.1 ± 0.7	111 ± 7	1.15 ± 0.15	17.1 ± 0.4
**26**				155 ± 7	0.54 ± 0.06	26.4 ± 0.6	125 ± 6	0.62 ± 0.13	22 ± 0.5	162 ± 9	0.81 ± 0.13	24 ± 0.6	117 ± 7	1.07 ± 0.12	17 ± 0.4
**29**				147 ± 7	0.44 ± 0.07	26.4 ± 0.5	113 ± 6	0.55 ± 0.07	22.3 ± 0.4	157 ± 8	1.06 ± 0.13	23.9 ± 0.7	105 ± 8	1.1 ± 0.14	17 ± 0.4
**33**				142 ± 7	0.41 ± 0.06	25.9 ± 0.4	137 ± 7	0.56 ± 0.07	22.9 ± 0.5	163 ± 8	0.73 ± 0.07	23.8 ± 0.7	94 ± 6	1.29 ± 0.19	16.9 ± 0.6
**36**				145 ± 8	0.36 ± 0.05	26 ± 0.5	116 ± 8	0.53 ± 0.1	23 ± 0.6	157 ± 9	0.62 ± 0.09	23.9 ± 0.7	106 ± 8	1.24 ± 0.14	16 ± 1.7

B -SK-N-BE(2)

**Diet**	**SD w/o CTx**			**SD**			**CR-SD**			**KD**			**CR-KD**		
day	BG	KB	BW	BG	KB	BW	BG	KB	BW	BG	KB	BW	BG	KB	BW
5	162 ± 12	0.5 ± 0.06	25.3 ± 1.2	169 ± 9	0.46 ± 0.06	23.6 ± 0.4	133 ± 8	0.71 ± 0.09	21.8 ± 0.5	177 ± 7	0.94 ± 0.1	22.9 ± 0.5	113 ± 11	1.76 ± 0.2	19.9 ± 0.4
8	164 ± 8	0.49 ± 0.1	25.8 ± 1.2	162 ± 7	0.44 ± 0.06	23.4 ± 0.4	148 ± 11	0.73 ± 0.07	21.1 ± 0.4	186 ± 9	1.12 ± 0.1	23 ± 0.4	135 ± 6	1.01 ± 0.13	18.9 ± 0.5
12	166 ± 8	0.5 ± 0.07	25.8 ± 1.3	164 ± 13	0.5 ± 0.07	23.4 ± 0.4	143 ± 12	0.57 ± 0.09	20.5 ± 0.6	162 ± 11	1.08 ± 0.12	23.6 ± 0.3	109 ± 7	1.28 ± 0.11	18.2 ± 0.5
15	167 ± 9	0.54 ± 0.09	26.5 ± 1.3	164 ± 8	0.56 ± 0.07	23.5 ± 0.3	145 ± 9	0.76 ± 0.11	20.3 ± 0.6	158 ± 6	0.89 ± 0.11	23.8 ± 0.4	124 ± 7	1.29 ± 0.09	18 ± 0.5
19	156 ± 9	0.51 ± 0.08	26.7 ± 1.2	164 ± 11	0.6 ± 0.06	24 ± 0.3	117 ± 10	0.78 ± 0.15	20.6 ± 0.5	162 ± 7	0.94 ± 0.07	24.1 ± 0.4	125 ± 8	1.39 ± 0.13	18.2 ± 0.7
22				158 ± 5	0.7 ± 0.09	23.9 ± 0.4	145 ± 11	0.62 ± 0.07	20.9 ± 0.5	164 ± 7	1.03 ± 0.09	24.2 ± 0.3	124 ± 7	1.35 ± 0.11	18.3 ± 0.7
26				151 ± 8	0.59 ± 0.09	24.2 ± 0.3	126 ± 8	0.69 ± 0.08	21.4 ± 0.5	165 ± 10	1.09 ± 0.08	24.3 ± 0.3	116 ± 7	1.08 ± 0.12	18.9 ± 0.8
29				166 ± 6	0.66 ± 0.07	23.9 ± 0.4	137 ± 10	0.71 ± 0.09	21.9 ± 0.5	166 ± 7	1.13 ± 0.07	24.3 ± 0.4	108 ± 7	1.37 ± 0.09	19.5 ± 0.6
33				145 ± 10	0.64 ± 0.09	24 ± 0.4	133 ± 5	0.71 ± 0.05	22.3 ± 0.6	174 ± 8	1.06 ± 0.07	24.3 ± 0.3	115 ± 9	1.08 ± 0.11	19.5 ± 0.6
36				156 ± 6	0.68 ± 0.09	24.2 ± 0.3	122 ± 8	0.69 ± 0.12	22.3 ± 0.7	161 ± 9	1.16 ± 0.08	24.4 ± 0.3	117 ± 7	1.42 ± 0.14	19.7 ± 0.6

Supplementary Table S2: Glucose ketone index (GKI) at each measured time point for different therapy groups

A-SH-SY5Y

day	Diet Group				
	SD w/o CTx	SD	CR-SD	KD	CR-KD
**5**	22.8	24.9	11.4	7.9	3.6
**8**	25.3	20.0	10.1	10.2	3.8
**12**	19.2	18.3	8.4	11.3	3.9
**15**	20.7	18.4	11.4	9.8	4.1
**19**	20.5	21.3	11.2	9.5	5.2
**22**		15.8	11.5	7.9	5.4
**26**		15.9	11.2	11.1	6.1
**29**		18.5	11.4	8.2	5.3
**33**		19.2	13.6	12.4	4.0
**36**		22.4	12.1	14.1	4.7
**Average**	**21.7**	**19.5**	**11.2**	**10.2**	**4.6**

B-SK-N-BE(2)

day	Diet Group				
	SD w/o CTx	SD	CR-SD	KD	CR-KD
**5**	18.0	23.5	10.5	10.9	3.7
**8**	22.8	22.5	11.7	9.4	7.5
**12**	18.4	18.2	15.9	9.0	5.0
**15**	18.5	18.2	11.5	11.0	5.7
**19**	17.3	15.2	9.3	10.0	5.3
**22**		12.5	13.4	9.1	5.3
**26**		16.8	11.7	9.2	6.4
**29**		15.4	10.9	8.4	4.6
**33**		13.4	10.5	9.7	6.4
**36**		14.4	11.3	8.1	4.6
**Average**	**19.0**	**17.0**	**11.7**	**9.5**	**5.5**

Supplementary Table S3

SH-SY5Y

i_1 (MCP)	0.23			
	i_2 (Diet, Morscher et al. 2015)	hypothetical $i_{1,2}$	calculated $i_{1,2}$	Ratio (calc./hypoth.)
CR-SD	0.52	0.12	0.11	0.96
KD	0.48	0.11	0.09	0.86
CR-KD	0.33	0.08	0.03	0.44

SK-N-BE(2)

i_1 (MCP)	0.07			
	i_2 (Diet, Morscher et al. 2015)	hypothetical $i_{1,2}$	calculated $i_{1,2}$	Ratio (calc./hypoth.)
CR-SD	0.55	0.04	0.02	0.61
KD	0.91	0.06	0.04	0.62
CR-KD	0.28	0.02	0.04	1.89

Supplementary Table S4: Ingredient list of the two diets

	**Standard Diet**	**Ketogenic Diet**
**Crude Nutrients**	**%**	%
Dry matter	87.8	98.0
Crude protein	19.0	15.9
Crude Fat	3.3	57.0
Crude fibre	4.9	4.6
Crude ash	6.4	5.2
N free extracts	54.2	15.5
Carbohydrates	41.3	11.1
Starch	36.6	0.1
Saccharose	4.7	11.0
**Fatty Acids**	%	%
short chain	–	0.19
medium chain	–	0.69
long chain	3.33	53.69
**Minerals**	%	%
Calcium	1.00	0.92
Phosphorus	0.70	0.61
Sodium	0.24	0.19
Magnesium	0.23	0.21
Potassium	0.92	0.95
**Amino Acids**	**%**	%
Lysine	1.10	1.32
Methionine	0.35	0.59
Met+Cys	0.70	0.86
Threonine	0.68	0.70
Tryptophan	0.25	0.21
Arginine	1.16	0.62
Histidine	0.45	0.48
Valine	0.90	1.11
Isoleucine	0.78	0.90
Leucine	1.33	1.58

Phenylalanine	0.87	0.82
Phe+Tyr	1.47	1.66
Glycine	0.82	0.34
Glutamic acid	3.97	3.58
Aspartic acid	1.66	1.18
Proline	1.28	1.82
Alanine	0.81	0.53
Serine	0.92	0.95
